# Bearing Fault Diagnosis under Variable Speed Using Convolutional Neural Networks and the Stochastic Diagonal Levenberg-Marquardt Algorithm

**DOI:** 10.3390/s17122834

**Published:** 2017-12-06

**Authors:** Viet Tra, Jaeyoung Kim, Sheraz Ali Khan, Jong-Myon Kim

**Affiliations:** School of Electrical, Electronics and Computer Engineering, University of Ulsan, 44610 Ulsan, Korea; traviet.vt@gmail.com (V.T.); kjy7097@gmail.com (J.K.); sherazalik@gmail.com (S.A.K.)

**Keywords:** acoustic emissions, bearing, fault diagnosis, convolutional neural networks, stochastic diagonal Levenberg-Marquardt algorithm, variable speed

## Abstract

This paper presents a novel method for diagnosing incipient bearing defects under variable operating speeds using convolutional neural networks (CNNs) trained via the stochastic diagonal Levenberg-Marquardt (S-DLM) algorithm. The CNNs utilize the spectral energy maps (SEMs) of the acoustic emission (AE) signals as inputs and automatically learn the optimal features, which yield the best discriminative models for diagnosing incipient bearing defects under variable operating speeds. The SEMs are two-dimensional maps that show the distribution of energy across different bands of the AE spectrum. It is hypothesized that the variation of a bearing’s speed would not alter the overall shape of the AE spectrum rather, it may only scale and translate it. Thus, at different speeds, the same defect would yield SEMs that are scaled and shifted versions of each other. This hypothesis is confirmed by the experimental results, where CNNs trained using the S-DLM algorithm yield significantly better diagnostic performance under variable operating speeds compared to existing methods. In this work, the performance of different training algorithms is also evaluated to select the best training algorithm for the CNNs. The proposed method is used to diagnose both single and compound defects at six different operating speeds.

## 1. Introduction

Rolling element bearings are extensively used in rotating machines to ensure their smooth operation. They are sturdy components with typically very long useful lives, yet they account for more than 51% of failures in induction motors alone [1]. Breakdown of critical equipment such as induction motors can lead to unscheduled and costly downtime for an entire production facility. Thus, the detection of incipient bearing defects is an important part of condition-based maintenance (CBM). Fault diagnosis and estimation has been done using different techniques in different domains [2,3,4,5]. Bearing faults have been diagnosed mostly using techniques, which diagnose bearing defects by analyzing different types of signals, such as the vibration acceleration signal of a bearing’s housing measured through accelerometers [6,7,8,9,10], the stator current of the induction motor [11,12,13], the acoustic emission (AE) signals [14,15,16,17], and the stray flux spectra [18]. Techniques that analyze the vibration acceleration signal and the motor stator current are effective in diagnosing bearing defects at high rotational speeds. However, at low rotational speeds, bearing defects, especially incipient defects, are more effectively diagnosed using AE-based methods, as they are sensitive to the low energy acoustic emissions released by a developing crack in the bearing even if it is sub-surface [19]. Hence, in this paper, AE signals are used to diagnose incipient bearing defects under variable operating speeds. The diagnosis of bearing defects under variable operating speeds is an important problem. Many studies [20,21] have considered similar problems in different contexts. For instance, the authors of [20,21] have studied the application of traditional vibration-based techniques for the diagnosis of various rotor faults in machines operating at different speeds and with different foundation supports.

AE-based methods mostly diagnose bearing defects either by using envelope analysis [22], or by constructing discriminative models for features extracted from the bearing fault signals using discriminative classifiers such as support vector machines (SVM) [23]. Envelope analysis-based methods diagnose bearing defects by looking for peaks at characteristic frequencies associated with each defect type in the power spectrum of the envelope signal [17,22,24,25]. However, these characteristic defect frequencies (CDFs) are functions of the bearing’s rotational speed [17,22,24,25], which renders these techniques ineffective under variable operating speeds. Similarly, feature extraction-based methods are also not effective in diagnosing bearing defects under variable operating speeds [26], as variations in the operating speed result in inconsistent features that yield poor discriminative models. Hence, these methods have predominantly been used to diagnose bearing defects under constant operating speeds. Moreover, since feature extraction-based methods use the statistical properties of the time and frequency domain AE signal, and the complex envelope signal; the diagnostic performance of these methods depends upon the quality of the extracted features. The selection of appropriate features requires both expert domain knowledge and feature selection algorithms to eliminate redundant and irrelevant features [14,23,27,28].

This paper presents a new approach to diagnosing bearing defects in order to address the two key limitations of existing AE-based techniques, i.e., they are not effective in diagnosing bearing defects under variable operating speeds and, in most cases, they require expert domain knowledge and special algorithms for the selection of optimal features. The proposed method automates the feature extraction process by using convolutional neural networks (CNNs), which automatically learn distinguishing features [29] from the spectral energy map (SEM) of an AE signal and use these features to diagnose various bearing defects. The proposed method is effective in diagnosing incipient bearing defects under variable operating speeds primarily due to two reasons. Firstly, the SEM of the AE signal is used as input to the CNNs. The SEM is a 2-D map that shows the relative distribution of the energy of the AE signal across different frequencies. As mentioned earlier, the CDFs and hence their harmonics are functions of the bearing’s rotational speed, and therefore, change with variation in bearing speed. However, variation in a bearing’s speed only translates these CDFs and their harmonics along the frequency axis but does not alter the strong correlation among nearby frequencies in the AE spectrum and hence, the relative distribution of energy among different frequencies; Secondly, the use of CNNs allows the proposed method to be effective under variable operating speeds as the classification performance of CNNs is not affected by the translation of its inputs, because CNNs exploit the local structure and correlation in the input data through their local receptive fields [29]. Thus, for a particular fault, the CNNs can effectively handle the translation effects on the SEMs that are induced by variations in the bearing speed, and therefore, can use these SEMs to diagnose different bearing defects under variable operating speeds.

This study uses the LeNet-5 architecture to automatically learn unique representations from the input SEMs for classifying various single and compound bearing defects. The performance of LeNet-5 is improved by finding the best training algorithm. A popular choice for training CNNs is the first order gradient descent (GD) algorithm, but it converges rather slowly and in the case of a non-quadratic error surface, the GD algorithm can get stuck in local minima [30]. The convergence speed of the GD is usually enhanced by using the Gauss-Newton algorithm, which approximates the error function using an appropriate quadratic function to determine the optimal step size in each direction [30]. However, the selection of an inappropriate quadratic function can cause the Gauss-Newton algorithm to diverge. The Levenberg-Marquardt algorithm is a robust training algorithm that combines the Gauss-Newton and the steepest descent methods to exploit the speed advantage of the former and the stability of the later. It yields better convergence results, even for complex non-quadratic error functions.

However, the Levenberg-Marquardt algorithm requires calculation of the Hessian matrix and its inverse, which is computationally very expensive, especially for large problems [31]. Therefore, we use the stochastic diagonal Levenberg-Marquardt (S-DLM) algorithm to train the CNNs, as it simplifies the computation of the Hessian and its inverse, and speeds up the training process as well. The S-DLM uses the diagonal terms of the Hessian matrix to compute the individual learning rates of all parameters of the CNNs before scanning through the training set [29]. Our empirical results indicate that CNNs converge quickly when trained using the S-DLM algorithm, thereby enhancing the performance of the proposed fault diagnosis system.

The main contributions of this paper include the following:

1. A new method is proposed to diagnose bearing defects under variable speed conditions using CNNs that automatically learn features from the input SEMs and use those features to diagnose various bearing defects.
2. Spectral energy maps (SEMs) of the AE signals are proposed for diagnosing bearing defects under variable speed conditions. For a given fault type, the SEMs show no significant variation irrespective of changes in the bearing speed, as demonstrated by the experimental results. Thus, the SEMs can serve as an ideal input to the CNNs for diagnosing bearing defects under variable operating speed.
3. This work also investigates various training algorithms for CNNs and proposes the use of S-DLM algorithm for training the CNNs as it results in faster convergence and a better diagnostic performance.

The rest of this paper is organized as follows: Section 2 presents the experimental testbed used in this study. Section 3 presents the proposed method, CNNs, the LeNet-5 architecture, and stochastic diagonal Levenberg-Marquardt algorithm. Section 4 provides details of the experiments carried out to validate the proposed method, whereas conclusions are provided in Section 5.

## 2. The Experimental Testbed and the Seeded Defect Acoustic Emission Data 

Acoustic emissions (AE) activity occurs whenever the rolling elements of a bearing pass over a localized defect in the bearing material [32]. The AE impulses are generated at CDFs, which primarily depend upon the location of the defect, operating speed, and bearing geometry that is defined by various parameters such as the number of rollers $\left(N_{rollers}\right)$, the contact angle (α), the roller diameter $\left(B_{d}\right)$, the pitch diameter $\left(P_{d}\right)$, and the shaft speed ($F_{shaft}$), as given in Equation (1). A defect on a bearing’s outer raceway generates high energy impulses at the ball pass frequency of the outer raceway (*BPFO*), whereas a defect on the inner raceway produces impulses at the ball pass frequency of the inner raceway (*BPFI*). Similarly, the ball spin frequency (*BSF*) is the rate at which impulses are produced when the bearing’s outer and inner raceways pass over a defect on the rolling element, and the fundamental train frequency (*FTF*) is the frequency with which the bearing cage rotates.

(1) $$\begin{array}{l} BPFI=\frac{N_{roller}\cdot F_{shaft}}{2}\left(1+\frac{B_{d}}{P_{d}}\cos\alpha \right)\\ BPFO=\frac{N_{roller}\cdot F_{shaft}}{2}\left(1-\frac{B_{d}}{P_{d}}\cos\alpha \right)\\ BSF=\frac{P_{d}\cdot F_{shaft}}{2\cdot B_{d}}\left(1-{\left(\frac{B_{d}}{P_{d}}\cos\alpha \right)}^{2}\right)\\ FTF=\frac{F_{shaft}}{2}\left(1-\frac{B_{d}}{P_{d}}\cos\alpha \right) \end{array}$$

**Figure 1:**
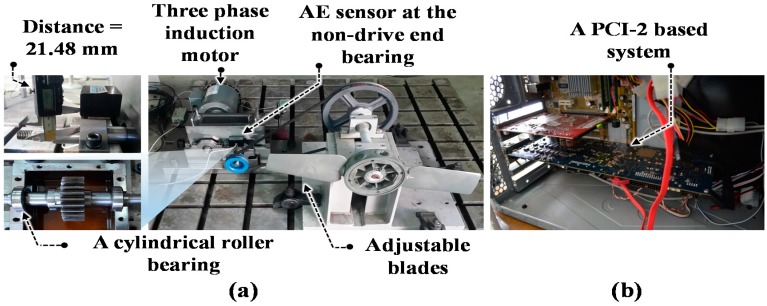
(**a**) The experimental testbed; (**b**) The data acquisition system.

The experimental testbed used to generate the acoustic emission (AE) data for seeded bearing defects at various operating speeds is shown in Figure 1a. It has two shafts, a drive end shaft (DES) and a non-drive end shaft (NDES); both of which are connected through a gearbox with a reduction ratio of 1.52:1. The shafts are fastened at both ends using rolling elements bearings (FAG NJ206-E-TVP2). A three-phase induction motor is used to power the DES at six different speeds, i.e., 250, 300, 350, 400, 450, and 500 revolutions per minute (r/min). A displacement transducer installed on the NDES is used to measure the operating speed. The AE data is collected for bearings fastened to the NDES using a wide-band AE sensor with an operating frequency ranging from 100 to 900 kHz, and a resonant frequency of 125 kHz [33]. The AE sensor is coupled to the bearing housing at a distance of 21.48 mm. These bearings are seeded with both single and compound defects of different sizes. A fan with adjustable blades is also coupled to the NDES to load the bearings. However, the effects of variations of the load are not considered in this study. The AE signals are logged at a sampling rate of 250 kHz using a PCI-2 data acquisition system, as shown in Figure 1b. A high sampling rate of 250 kHz is used as the AE activity mostly occurs in the ultrasonic range [32].

**Figure 2:**
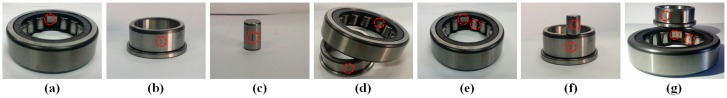
The single and compound seeded bearing defects with a crack length, width, and depth of 3, 0.35, and 0.3 mm, respectively: (**a**) BCI; (**b**) BCO; (**c**) BCR; (**d**) BCIO; (**e**) BCIR; (**f**) BCOR; and (**g**) BCIOR.

The AE signals are recorded at six different speeds for a healthy bearing and bearings seeded with seven types of defects, i.e., outer raceway crack (BCO), inner raceway crack (BCI), roller crack (BCR), inner and outer raceway cracks (BCIO), outer and roller cracks (BCOR), inner and roller cracks (BCIR), and inner, outer, and roller cracks (BCIOR), as shown in Figure 2. Each AE signal is recorded for a duration of 5 s, and hence carries information for at least 40 bearing revolutions. For each fault type and at each operating speed, a total of 90 AE signals are recorded.

## 3. The Proposed Method for Diagnosing Bearing Defects under Variable Speeds

**Figure 3:**
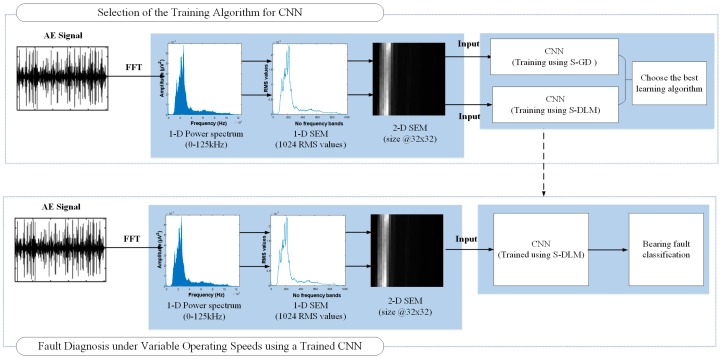
The proposed method for the diagnosis of single and compound bearing defects using acoustic emission signals under variable operating speeds.

The proposed method for bearing fault diagnosis under variable operating speeds is illustrated in Figure 3. It uses CNNs because they are very good at automatically learning distinctive features from their inputs that result in the best classification performance. The proposed method uses a representation learning technique, such as CNN, because it is difficult to manually design features of the AE signal that would be effective in diagnosing both simple and compound bearing defects under variable operating speeds. This point is further illustrated in Section 4.3. CNNs are very good at learning distinctive representations of their inputs, nevertheless, they come with certain caveats; first, the input to the CNN must have strong spatial correlation, and second, it must have reasonable size or dimensions, i.e., input with large dimensions would require more training data and prolonged training times that would restrict their practical use [34]. A five second AE signal that is sampled at 250 KHz has 1.25 × 10^6^ samples, and hence cannot be used as input to the CNN. Therefore, just as important it is to use CNNs, an even more important concern is to decide the appropriate type and size of input for the CNN that can be effective in diagnosing bearing defects under variable operating speeds. We propose to use 2-D spectral energy maps or SEMs as inputs to the CNN. The intuition behind using SEMs as inputs to the CNNs is that for a given fault type, the shape of the AE power spectrum and SEM doesn’t change much with changes in the rotational speed of the bearing, and hence SEMs can be used as inputs to the CNNs to diagnose bearing defects when there are variations in rotational speed. This is further illustrated in Section 4.

In the proposed method, the raw AE signals are converted into 2-D SEMs. First, the fast Fourier transform (FFT) of the raw AE signals is computed to get their frequency content. As discussed earlier, for a given fault type, the shape of the AE spectrum is not significantly altered by changes in the bearing’s rotational speed. The AE spectrum has 1.25 × 10^5^ frequency components and it is also not a suitable input to the CNN. Therefore, the AE spectrum is divided into 2^10^ frequency bands and for each of these bands the root mean square (RMS) frequency is calculated, resulting in a 1-D SEM with RMS values for 1024 frequency bands. These RMS values approximate the energy carried by each band. The RMS values in the 1-D SEM are stacked on top of each other to create a 2-D SEM of size 32 × 32. The 2-D SEM has reasonable dimensions and good spatial correlation making it a suitable input for the CNN. These 2-D SEMs are then used to train the CNNs using different training algorithms as discussed in Section 4. The CNNs trained with the best training algorithm, i.e., S-DLM are then used for diagnosing bearing defects under variable operating speeds.

### 3.1. CNNs and the LeNet-5 Architecture

**Figure 4:**
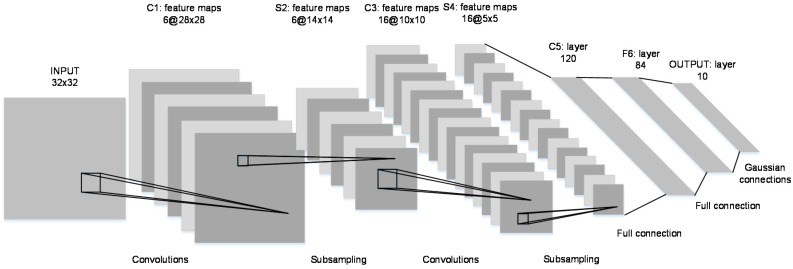
The LeNet-5 Architecture, a convolutional neural network.

CNNs are feedforward artificial neural networks (ANNs), which have been widely used for image classification, optical character recognition, and video analysis where it is difficult to design optimal features and the scaling and distortion of inputs can significantly degrade the classification performance [35]. CNNs utilize local connections (instead of fully-connected layers), weight sharing, and spatial or temporal sub-sampling to attain invariance to shifting, scaling, and distortion in their inputs [29]. Figure 4 shows the architecture of a typical CNN, the LeNet-5, which was initially proposed for character recognition [29]. It is different from typical ANNs in that the consecutive layers are not fully connected. Rather, every unit in a particular layer is connected to a small neighborhood of units or a local receptive field in the preceding layer. The neurons in a particular layer extract simple features from the receptive fields in the previous layers, and neurons in the subsequent layers combine these simple features to learn higher-order representations of the input. A distortion or shift in the inputs alters the position of these simple features. Therefore, CNNs use a weight-sharing mechanism to become invariant to shifts and distortions in the inputs. As a result, units with local receptive fields in separate locations share identical weight vectors, forcing them to perform the same operation and hence, extract the same features from the entire input field. These units thus construct a feature map for the entire input. Using different weight vectors and hence, different operations, a convolutional layer constructs several feature maps of the input.

### 3.2. Training CNNs Using the Stochastic Diagonal Levenberg-Marquardt Algorithm

CNNs are trained using learning algorithms such as the Levenberg-Marquardt algorithm, which determines the optimal network parameters, i.e., weights and biases. These algorithms are locally adaptive, as they determine the learning rates for each weight and bias in the network by considering both the gradient and curvature of the error function [31]. The weight update for a second order method such as Newton’s method is calculated as follows [36]:

(2) $$\Delta w=\eta_{g}{\left(\frac{\partial^{2}E}{\partial w^{2}}\right)}^{-1}\frac{\partial E}{\partial w}=\eta_{g}H{\left(w\right)}^{-1}\frac{\partial E}{\partial w},$$

where $\eta_{g}\in (0,1)$ is the global learning rate and ${\left(\partial^{2}E/\partial w^{2}\right)}^{-1}$ is the inverse of Hessian matrix, which is used to compute the individual or local learning rates. The computation of the inverse of the Hessian matrix is impractical for large neural networks since it requires $O(N^{3})$ operations for each update to *N* network parameters [31].

Therefore, the stochastic diagonal Levenberg-Marquardt method (S-DLM) is proposed to update the network parameters. It approximates the Hessian matrix only by its diagonal terms and drops the off-diagonal elements. The S-DLM requires O(N) operations for each update to the *N* network parameters, making it suitable for large networks. The individual learning rate for a given weight, $w_{ij}$, is determined as follows [37]:

(3) $$\eta_{ij}=\frac{\eta_{g}}{\left(\frac{\partial^{2}E}{\partial w_{ij}{}^{2}}\right)+\mu }$$

The adjustment $\Delta w_{ij}(n)$ to the weight $w_{ij}$ is calculated using Equation (4).

(4) $$\Delta w_{ij}=\frac{\eta_{g}}{\left(\frac{\partial^{2}E}{\partial w_{ij}^{2}}\right)+\mu }\left(\frac{\partial E^{p}}{\partial w_{ij}}\right),$$

Here, $h_{kk}=(\partial^{2}E/\partial w_{ij}^{2})$ estimates the second order derivative of the *k*-th diagonal element of the Hessian matrix with respect to the weight $w_{ij}$. The parameter μ is used to restrict the step size from becoming too large when the second order derivative becomes too small. The instantaneous gradient of the synaptic weight $\left(\partial E^{p}/\partial w_{ij}\right)$ can be efficiently calculated through backpropagation using the stochastic gradient descent method, which is explained in detail in [34]. The running estimate of the second order derivative $\partial^{2}E/\partial w_{ij}^{2}$ over the training samples is computed as follows:

(5) $${\left(\frac{\partial^{2}E}{\partial w_{ij}^{2}}\right)}_{new}=\left(1-\gamma \right){\left(\frac{\partial^{2}E}{\partial w_{ij}^{2}}\right)}_{old}+\gamma \left(\frac{\partial^{2}E^{p}}{\partial w_{ij}^{2}}\right),$$

where γ is a constant that determines the amount of memory being used. The computational cost of the second order derivatives given in Equation (5) is almost the same as that of the gradient in a standard back-propagation pass, except that the weighted sums use the square of the weights [37,38]. The instantaneous second order derivatives of the diagonal elements relative to the weights in the CNNs can be calculated by back-propagating the diagonal Hessian [31,37,38].

#### 3.2.1. Fully Connected Layers

The S-DLM algorithm requires that the error be calculated for every input pattern using the squared-error loss function. For the *p*th pattern, in a multiclass problem with *c* classes, the error is given by Equation (6),

(6) $$E^{p}=\frac{1}{2}{\sum_{k=1}^{c}\left(d_{k}^{p}-o_{k}^{p}\right)}^{2},$$

where $d_{k}^{p}$ is the *k*th component of the target, which corresponds to the *p*th pattern and $o_{k}^{p}$ is the value of the *k*th output for the *p*th input pattern. Let ℓ and *L* denote the current and output layers, respectively. Layer ℓ calculates its output by applying an activation function *f* to the sum of the dot product of the weight and input vectors, and the bias vector, as given in Equation (7), where *W* and *b* are the weight and bias vectors, respectively, and *x* is the input vector.

(7) $$x^{\ell }=f(y^{\ell }),\begin{array}{ccc} & with & y^{\ell } \end{array}=W^{\ell }x^{\ell -1}+b^{\ell }$$

Using the rules for the back-propagation of the diagonal Hessian in neural networks [31], the square of the local gradient is calculated as follows:

(8) $${\left(\delta^{\ell }\right)}^{2}=\frac{\partial^{2}E^{p}}{\partial {\left(y^{\ell }\right)}^{2}}={\left({\left(W^{\ell +1}\right)}^{T}\right)}^{2}{\left(\delta^{\ell +1}\right)}^{2}\circ {\left(f^{\prime }(y^{\ell })\right)}^{2},$$

where “∘” denotes element-wise multiplication. For the output layer neurons, the square of the local gradient will take a slightly different form as follows:

(9) $${\left((\delta^{L})\right)}^{2}={\left(f^{\prime }(y^{L})\right)}^{2}$$

Finally, the delta rule is used to calculate the squares of the weight and bias gradients for a given neuron in the fully connected layer of the CNNs using Equations (10) and (11), respectively.

(10) $$\frac{\partial^{2}E^{p}}{\partial {\left(W^{\ell }\right)}^{2}}={\left(x^{\ell -1}\right)}^{2}{\left({\left(\delta^{\ell }\right)}^{T}\right)}^{2}$$

(11) $$\frac{\partial^{2}E^{p}}{\partial {\left(b^{\ell }\right)}^{2}}=\frac{\partial^{2}E^{p}}{\partial {\left(y^{\ell }\right)}^{2}}{\left(\frac{\partial y^{\ell }}{\partial b^{\ell }}\right)}^{2}={\left(\delta^{\ell }\right)}^{2}$$

#### 3.2.2. Convolution Layers

Similarly, the square of the local gradient can be calculated for the convolution layers. Let j denote the current feature map. Then, for the convolution layer ℓ, the square of the local gradient is given by Equation (12) [34].

(12) $${\left(\delta_{j}^{\ell }\right)}^{2}={\left(\beta_{j}^{\ell +1}\right)}^{2}({\left(f^{\prime }(y_{j}^{\ell })\right)}^{2}\circ up{\left(\delta_{j}^{\ell +1}\right)}^{2}),$$

Here, up(.) represents the up-sampling operation, which copies each input *n* times in the vertical and horizontal directions of the output. The up-sampling factor *n* must be equal to the sub-sampling factor of the sub-sampling layer. Using the square of the local gradient for each feature map, the square of the bias gradient can be computed by summing the local gradient over all the entries, as given by Equation (13). The square of the kernel gradients can be determined using the recurrence relation in Equation (14).

(13) $$\frac{\partial^{2}E^{p}}{\partial {\left(b_{j}^{\ell }\right)}^{2}}=\sum_{u,v}(\delta_{j}^{\ell }{)}^{2}{}_{uv}\;,$$

(14) $$\frac{\partial^{2}E^{p}}{\partial {\left(k_{ij}^{\ell }\right)}^{2}}=\sum_{u,v}(\delta_{j}^{\ell }{)}^{2}{}_{uv}{\left(p_{i}^{\ell -1}\right)}^{2}{}_{uv}\;,$$

Here, ${\left(p_{i}^{\ell -1}\right)}_{uv}$ is the patch in $x_{i}^{\ell -1}$, which is used to compute the element at position (u,v) in the output feature map $x_{j}^{\ell }$ through convolution with the kernel $k_{ij}^{\ell }$.

#### 3.2.3. Sub-Sampling Layers

Similarly, after finding the square of the local gradient ${\left(\delta_{j}^{\ell }\right)}^{2}$ for neurons in the sub-sampling layer, the gradient of the bias *b* and weight β can be obtained using Equations (15) and (16), respectively.

(15) $$\frac{\partial^{2}E^{p}}{\partial {\left(b_{j}^{\ell }\right)}^{2}}=\sum_{u,v}(\delta_{j}^{\ell }{)}^{2}{}_{uv}$$

(16) $$\frac{\partial E^{p}}{\partial {\left(\beta_{j}^{\ell }\right)}^{2}}={\sum_{u,v}\left((\delta_{j}^{\ell }\right)}^{2}\circ down{\left(x_{j}^{\ell -1}\right)}^{2}{}_{uv})$$

In Equation (16), down(.) represents the sub-sampling operation. Since the second order properties of the error function change rather slowly and are mostly determined by the structure of the network and not the statistical nature of the training data [29], in practice, the second order derivative in Equation (5) needs to run only on a small random subset of the training data before each pass of the learning algorithm [29].

## 4. Experimental Results and Discussion

### 4.1. Configuration of the Fault Signatures’ Pool

The performance of the proposed method is confirmed using two experiments. The first experiment verifies the effectiveness of the stochastic diagonal Levenberg-Marquardt (S-DLM) algorithm by comparing it with the stochastic gradient descent (S-GD) algorithm. The second experiment compares the performance of the proposed method with traditional AE-based methods for bearing fault diagnosis under variable operating speeds. 

The first experiment uses three datasets. Each dataset contains AE signals for a defect-free bearing and bearings seeded with seven types of localized defects, as shown in Figure 2. The dimensions of the defects and the operating speeds at which the AE signals are recorded are given in Table 1. For each operating speed, 90 AE signals with a duration of five seconds each are recorded for each fault type. Hence, each dataset contains a total of 720 AE signals. Each dataset is divided into training and testing subsets containing half of the total, i.e., 360 AE signals each.

Two datasets are used in the second experiment to verify the diagnostic performance of the proposed method under variable operating speeds. Both datasets contain AE signals for bearings seeded with the seven types of faults shown in Figure 2 and a defect free bearing, i.e., a total of eight different bearing conditions. The datasets consider crack sizes of 3 mm and 12 mm. Hence, each dataset has signals for eight bearing conditions, which are recorded at six operating speeds of 250, 300, 350, 400, 450, and 500 revolutions per minute (r/min). For each dataset, the AE data is divided into training and testing subsets, as shown in Table 2. The training subset includes AE signals acquired at speeds of 300, 400, and 500 r/min, while the testing subset includes the AE signals recorded at speeds of 250, 350, and 450 r/min. For each operating speed, 90 AE signals for each bearing condition are recorded for a duration of five seconds each. Hence, each dataset contains $N_{RPM}\times N_{Classes}\times N_{Signals}$ or 4320 AE signals, where $N_{RPM}$ is the number of operating speeds for which the AE signals are recorded ($N_{RPM}=6$), $N_{Classes}$ is the total number of defect types or bearing conditions ($N_{Classes}=8$), and $N_{Signals}$ is the total number of AE signals recorded for each bearing condition at each shaft speed ($N_{Signals}=90$).

**Figure 5:**
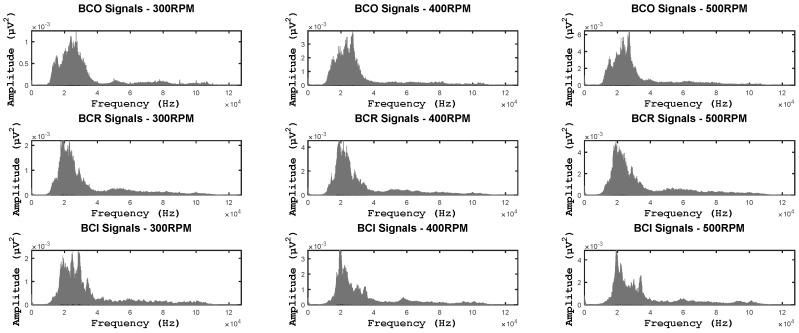
The Single-band power spectra of fault signals at different operating speeds.

The CNNs were designed to work on image data, which is intrinsically 2-D. On the other hand, we diagnose bearing defects using AE signals, which are 1-D in nature and are captured at very high sampling rates. Even if we stack the 1-D AE signals to create a 2-D input for the CNNs, mining the raw AE signal for distinctive features would require very large CNNs. Hence, as discussed earlier the raw AE signals are converted into an equivalent 2-D representation, which shows the distribution of energies in various frequency bands of the AE signal, i.e., the spectral energy map or SEM, which is then used as the input to the CNNs. The SEM is generated by first multiplying the AE signal by a Hanning window function and then computing its FFT, which is shown in Figure 5.

**Figure 6:**
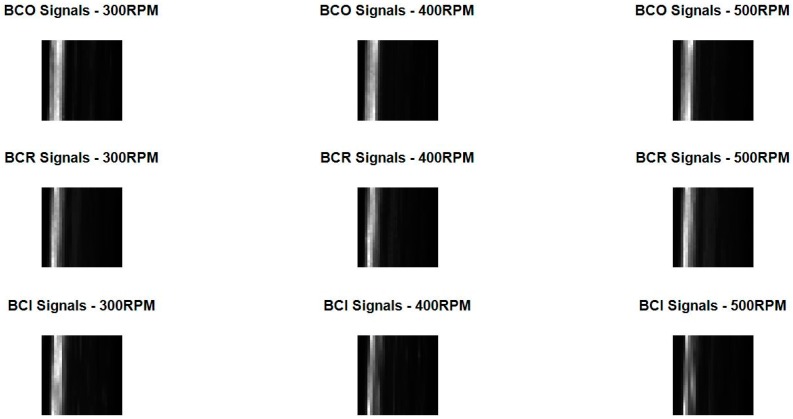
The spectral energy maps or SEMs for three different fault types at three different operating speeds. These SEMs are used as input by the CNNs.

The spectrum of the AE signal is then split into an appropriate number of frequency bands and the root mean square (RMS) value of each of these bands is calculated. The RMS value gives an approximation of the energy carried by each band [39]. These RMS values are arranged in the form of a 2-D array, which shows the distribution of energies across the entire spectrum of the AE signal. This 2-D array or SEM of the AE signal, examples of which are shown in Figure 6, is then used as the input for the CNNs.

### 4.2. Efficacy of the Stochastic Diagonal Levenberg-Marquardt Algorithm

As discussed in Section 3.2, the second order derivative in Equation (5) can be estimated without scanning the entire training set. Rather, it can be approximated using only a small subset or mini-batch of the training data before each iteration of the learning algorithm. Therefore, the second order derivative in Equation (5) can be estimated as follows:

(17) $$\frac{\partial^{2}E}{\partial w_{ij}^{2}}=\frac{1}{P}\sum_{p=1}^{P}\frac{\partial^{2}E^{p}}{\partial w_{ij}^{2}},$$

where *P* is the number of samples used to estimate the second order diagonal derivative. The approximation of the second order derivative in Equation (17) is not affected by the choice of the mini-batch [29]. Due to this property, such a training algorithm is labeled as the Mini-batch diagonal Levenberg-Marquardt algorithm (M-DLM).

This study compares the performances of three training algorithms for CNNs under the same conditions: the stochastic diagonal Levenberg-Marquardt algorithm (S-DLM), the stochastic gradient descent algorithm (S-GD), and the mini-batch diagonal Levenberg-Marquardt (M-DLM) algorithm. The performance of the algorithms is determined in terms of the average training time of a single epoch, convergence, and classification accuracy for the datasets listed in Table 1.

#### 4.2.1. Average Training Time of a Single Training Epoch

**Figure 7:**
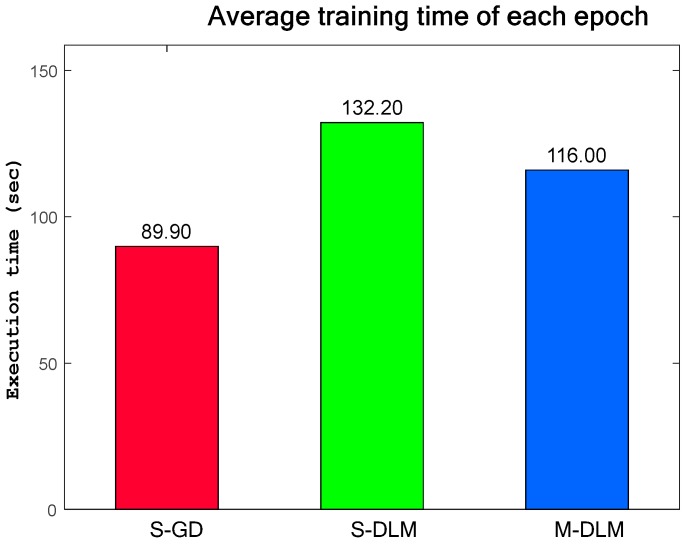
The average execution time of a single training epoch for various learning algorithms.

In comparison to S-GD, the S-DLM and M-DLM algorithms are not expected to decrease the training time as they must estimate the Hessian, which is time consuming. The results in Figure 7 affirm this notion as the S-GD requires the least amount of time to train the CNNs. The S-DLM (equivalent to M-DLM with a mini-batch size of 1) is the slowest training algorithm as it requires scanning through the entire training set before each learning iteration. The M-DLM requires more time compared to S-GD, but less than S-DLM since it uses only a small number of training samples and also omits the memory constant γ, further simplifying its computation. The training time of the M-DLM algorithm can be further reduced by either decreasing the mini-batch size or the frequency of re-estimating the second order derivative. In this test, the M-DLM algorithm uses a mini-batch of 50 training samples and re-estimates the second order derivative after each epoch. The results are generated using Matlab on a general purpose computing platform with an overclocked 3.3 GHz Intel Core i5-2500 CPU.

#### 4.2.2. Convergence of the Learning Algorithms

**Figure 8:**
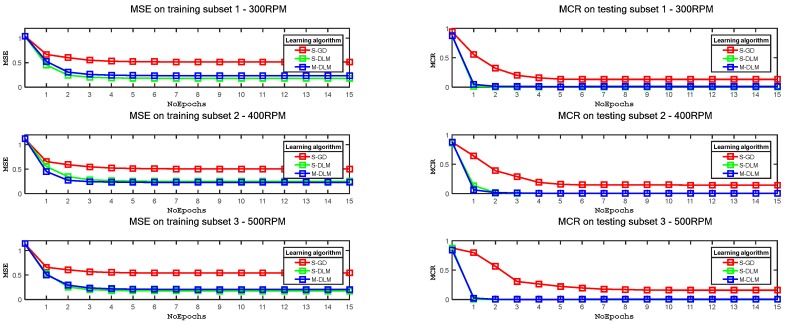
Training mean square error and testing misclassification error for dataset 1 using S-GD, S-DLM, M-DLM learning algorithms.

The learning performance of these algorithms is measured primarily by examining the convergence speed of these algorithms. Figure 8 shows both the mean square error (MSE) plots for the training samples and the misclassification rate (MCR) plots for the testing samples of the datasets given in Table 1. The learning curves, i.e., MSE plots, indicate that the S-DLM and M-DLM algorithms train the CNNs better than the S-GD, as these algorithms utilize the second order curvature information of the error function. The S-DLM and M-DLM algorithms enable the CNNs to reach the global minimum in just 2 epochs. In contrast, the S-GD yields relatively poor convergence performance. The learning performance of these algorithms is also examined through their MCR on the testing subset. The MCR plots in Figure 8 clearly indicate that the S-DLM and M-DLM algorithms enable the CNNs to learn the discriminative model for the training data better than the S-GD and thus, yield better MCR values for the testing subset, which are close to zero after only two training epochs.

#### 4.2.3. Classification Accuracy of Different CNNs

As mentioned in Section 4.1, two experiments are performed; the first experiment is performed to determine the best training algorithm using the datasets described in Table 1, while the second experiment is performed to compare the diagnostic performance of the proposed approach with existing techniques using the datasets described in Table 2. In both the experiments the average classification accuracy (*ACA*) is computed using Equation (18). The results of the first experiment, i.e., the *ACA* and average sensitivity values for the CNNs trained using the different training algorithms, are given in Table 3.

(18) $$ACA=\frac{\sum_{N_{classes}}N_{TP}}{N_{testdata}}\times 100\left(\%\right)\;,$$

Here, $N_{testdata}$ is the total number of samples used to test the classification accuracy of the proposed method, $N_{classes}$ is the total number of defect types, and $N_{TP}$ is the number of data points in class i, which are correctly classified as class i. The *sensitivity*, which is a useful metric for evaluating the diagnostic performance of the proposed method in relation to each bearing condition, is defined as follows:

(19) $$Sensitivity=\frac{N_{TP}}{N_{TP}+N_{FN}}\times 100\left(\%\right)\;,$$

where $N_{FN}$ is the number of data points in class i, which are classified incorrectly. In the first experiment the average classification accuracy is calculated after performing 20 runs of the training and testing processes. In each run the CNN is trained using a randomly selected training set and then its performance is tested on a randomly selected test set. Thus, each value of the *sensitivity* and average classification accuracy, given in Table 3, is an average that is calculated after performing 20 runs of the training and testing processes. The results shown in Table 3 demonstrate that the S-DLM and M-DLM algorithms are more powerful than the traditional S-GD algorithm for training CNNs, and can yield better diagnostic performance.

### 4.3. Performance Evaluation of the Proposed Method for Bearing Fault Diagnosis under Variable Operating Speeds

**Figure 9:**
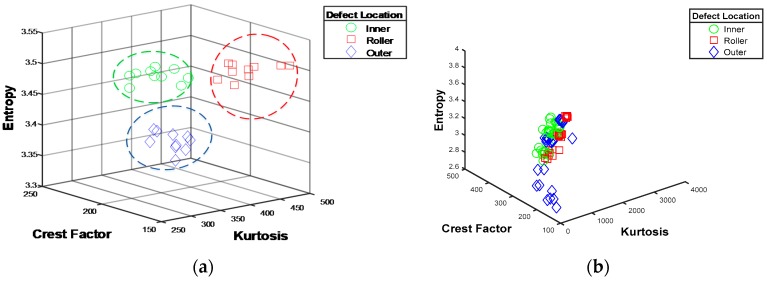
Cluster plots for features of AE signals in 3-d feature space at (**a**) Constant speed for BCI, BCR and BCO faults; (**b**) Variable speed for BCI, BCR and BCO faults.

The key difference between CNNs-based techniques and the traditional analysis-based methods is the manner of extracting features. The diagnostic performance of conventional methods depends on the extraction and selection of the best feature set, whereas CNNs can automatically learn the optimal features from their inputs. Traditional AE-based methods for bearing fault diagnosis have mostly been tested under constant operating speeds because variation of the speed results in unstable and inconsistent features, which significantly degrade their diagnostic performance. This is illustrated in Figure 9, which shows the distribution of features extracted from the AE signals for BCI, BCR and BCO faults. When there are no variations in speed, the extracted features can usually be easily separated as shown in Figure 9a. However, variations in speed can lead to overlapping in the feature space, as shown in Figure 9b, which can degrade the diagnostic performance of traditional AE-based methods.

The proposed method, however, uses the SEMs and CNNs to diagnose bearing defects under variable operating speeds and therefore, can effectively diagnose the eight types of bearing conditions considered in this study. The proposed method is compared to AE-based methods used for bearing fault diagnosis [14,15]. In these methods, various statistical measures of the time and frequency domain AE signals are used as features [14,15], along with statistical quantities calculated through complex envelope analysis [14]. Moreover, before feature extraction, the authors in [15] also consider the subband analysis of the AE signals using the discrete wavelet packet transform (DWPT). The extracted features are then evaluated and selected using feature selection algorithms such as outlier-insensitive hybrid feature selection (OIHFS) [14]. Finally, the selected features are used to detect bearing defects using classifiers such as K-NN [14] and SVM [15].

Table 4 presents the results of the second experiment, wherein the diagnostic performance of the proposed method and the techniques previously proposed in [14,15] is compared using the datasets described in Table 2. In the second experiment the CNN is trained using the best learning algorithm as determined in the first experiment, i.e., S-DLM. The diagnostic performance of all the methods is measured in terms of the ACA and sensitivity. The CNN is trained using AE signals for 300, 400 and 500 r/min, which are all packed into a single dataset; whereas, it is tested using AE signals for 250, 350, and 450 r/min, all packed into a single dataset. Thus, each value of the sensitivity and average classification accuracy, given in Table 4, is an average that was calculated after repeating the training and testing processes for at least 20 times. It is clear from the results given in Table 4 that the proposed method delivers better diagnostic performance than the previous methods [14,15]. The average classification accuracy of the proposed method is 98.47%, which shows significant improvement when compared to the methods proposed previously [14,15], i.e., by 47.09% and 23.03%, respectively.

## 5. Conclusions

The performance of traditional AE based methods for the diagnosis of incipient bearing defects relies on the quality of the features used to construct discriminative fault models, or on the detection of peaks at the characteristic frequencies, associated with each type of localized bearing defect, in the envelope power spectrum of the AE signals. These approaches can experience certain limitations, especially when it is difficult to design discriminative features or the features become inconsistent with changes in the operating conditions, such as, the bearing’s rotational speed. Since the characteristic defect frequencies are functions of the bearing’s rotational speed, therefore, variations in the bearing’s rotational speed would smear these frequencies across the envelope power spectrum making their detection very challenging. The purpose of this study was to devise a data-driven approach that can be used to diagnose single and compound defects in bearings, under variable operating speeds using AE signals, and without requiring any additional sensors or measurements. It was hypothesized that the variation of a bearing’s speed would not alter the overall shape of the AE spectrum, rather it may only scale and translate it. Thus, for a given defect type, the shape of the AE spectrum would not change significantly at different rotational speeds and, therefore, can be used for its detection. Hence, we proposed to diagnose bearing defects under variable operating speeds using CNNs due to their ability to automatically learn the optimal features from their inputs, and their invariance to any shifts and distortions in the inputs. In the scenario considered in this study, these distortions in inputs can be caused by variations in a bearing’s speed. The diagnostic performance of CNNs depends upon the selection of appropriate inputs and training algorithm. This study further proposed to transform the AE spectrum into SEM, which is a two-dimensional map that shows the distribution of energy across different bands of the AE spectrum. The SEMs were then used as input to a CNN. Another major concern in deep learning techniques, such as CNNs, is the selection of the appropriate training algorithm. Our results indicated that CNNs trained using the S-DLM algorithm yield the best diagnostic performance and require less training time. The experimental results confirmed our hypothesis, that the SEMs can be used to diagnose bearing defects under variable operating speeds. The proposed method diagnosed both single and compound bearing defects with 47% and 23% more accuracy compared to two state-of-the-art methods. 

## Figures and Tables

**Table 1:** Description of the datasets used to evaluate the proposed method in experiment 1.

*fs* = 250 kHz		Operating Speed (r/Min) ^1^	Crack Size		
			Length (mm)	Width (mm)	Depth (mm)
Dataset 1	Training set	300	3	0.35	0.30
	Testing set	300		0.35	0.30
Dataset 2	Training set	400	3	0.35	0.30
	Testing set	400		0.35	0.30
Dataset 3	Training set	500	3	0.35	0.30
	Testing set	500		0.35	0.30

^1^ For each bearing condition, i.e., a defect-free bearing and 7 defective bearings, 90 five-second AE signals were recorded at each r/min.

**Table 2:** Description of the datasets used to evaluate the proposed method in Experiment 2.

*fs* = 250 kHz		Operating Speed (r/Min) ^1^	Crack Size		
			Length (mm)	Width (mm)	Depth (mm)
Dataset 4	Training set	300, 400, 500	3	0.35	0.30
	Testing set	250, 350, 450		0.35	0.30
Dataset 5	Training set	300, 400, 500	12	0.49	0.50
	Testing set	250, 350, 450		0.49	0.50

^1^ For each bearing condition, i.e., a defect-free bearing and 7 defective bearings, 90 five-second AE data samples were obtained at each r/min.

**Table 3:** The results of Experiment No. 1 in terms of the average classification accuracy and sensitivity for each bearing defect type under variable speed conditions using different training algorithms for the CNNs.

Datasets	Learning Algorithm	Average Sensitivity of Each Fault Type								ACA (%)
		BCI	BCO	BCR	BCIO	BCIR	BCOR	BCIOR	BNC	
Dataset 1	S-GD	100	91.11	100	51.11	60	91.11	100	100	86.66
	S-DLM	100	95.55	100	100	100	100	100	100	99.44
	M-DLM	100	93.33	100	100	100	100	100	97.77	98.88
Dataset 2	S-GD	95.55	100	100	100	86.66	4.44	97.77	100	85.55
	S-DLM	100	100	100	100	100	97.77	100	100	99.72
	M-DLM	97.77	100	100	100	100	97.77	100	100	99.44
Dataset 3	S-GD	100	6.66	100	100	100	75.55	100	91.11	84.16
	S-DLM	100	100	100	100	100	100	100	100	100
	M-DLM	97.77	100	100	100	100	100	100	100	99.72

**Table 4:** The results of Experiment No. 2 in terms of average classification accuracy (ACA) and sensitivity for each bearing defect type under variable speed conditions using the proposed method and two state-of-the-art methods.

Datasets	Methodologies	Average Sensitivity for Each Fault Type								ACA (%)
		BCI	BCO	BCR	BCIO	BCIR	BCOR	BCIOR	BNC	
Dataset 4	[9]	19.62	47.40	75.18	17.03	59.62	30.74	10	3.33	32.87
	[10]	11.11	13.33	100	100	97.77	97.77	0	0	52.5
	Proposed	66.66	100	100	100	89.25	99.25	99.25	99.62	94.25
Dataset 5	[9]	7.03	70	66.66	79.62	5.92	44.81	74.07	62.96	51.38
	[10]	100	100	97.77	97.77	100	100	0	0	74.44
	Proposed	100	100	91.85	98.14	99.25	99.25	100	99.25	98.47
